# Antimicrobial prescribing quality in Australian emergency departments: an analysis of the Hospital NAPS data set

**DOI:** 10.1017/ash.2024.483

**Published:** 2025-01-17

**Authors:** Lenna Zosky-Shiller, Karin Thursky, Sarah Park, Rodney James, Lisa Hall, Caroline Chen, Courtney Ierano

**Affiliations:** 1 Melbourne Medical School, University of Melbourne, Melbourne, VIC, Australia; 2 National Centre for Antimicrobial Stewardship, Department of Infectious Diseases, University of Melbourne, Melbourne, VIC, Australia; 3 Royal Melbourne Hospital Guidance Group, Melbourne Health, Melbourne, VIC, Australia; 4 School of Public Health, The University of Queensland, Brisbane, QLD, Australia

## Abstract

**Objective:**

To analyze antimicrobial prescribing practices in Australian emergency departments (ED), identifying prescribing areas requiring improvement. This aims to inform antimicrobial stewardship (AMS) strategies to enhance antimicrobial prescribing quality.

**Design:**

Retrospective analysis of the Hospital National Antimicrobial Prescribing Survey (NAPS) data set.

**Setting:**

EDs in public and private Australian hospitals (n = 652).

**Participants:**

Hospitals (n = 652) that participated in the Hospital NAPS from 2013 to 2022.

**Methods:**

Data were collected by trained auditors from participating hospitals with the use of a standardized auditing tool, the Hospital NAPS. Data from 2013 to 2022 were analyzed descriptively. Variables assessed included guideline compliance and appropriateness by antimicrobial and indication, and reasons for inappropriateness.

**Results:**

There were 3,098 antimicrobial prescriptions from EDs included for analysis. Guideline compliance (63.5%) and appropriateness (70.4%) in EDs were lower compared to overall prescribing practices from all departments. The most commonly prescribed antimicrobial was ceftriaxone (16.9%, n = 523), and the most common indication was empiric prescribing for community-acquired pneumonia (16.0%, n = 497). Amoxicillin-clavulanic acid (53.2%, n = 99), and acute exacerbation of chronic obstructive pulmonary disease (54.3%, n = 57), were the antimicrobial and indication with the lowest rates of appropriateness respectively. Ceftriaxone prescribing also had a low rate of appropriateness (62.3%, n = 326). Selection of antimicrobials with too broad of a spectrum was the most common reason for inappropriateness (40.2%).

**Conclusion:**

Antimicrobial prescribing quality in EDs warrants improvement. Recommended targets for AMS interventions are the excessive and inappropriate use of broad-spectrum antimicrobials such as ceftriaxone and amoxicillin-clavulanic acid in common respiratory and urinary tract infections.

## Introduction

Each year, millions of patients are seen in emergency departments (EDs) globally with bacterial infections.^1,2^ Therefore, to prevent serious consequences such as sepsis, appropriate antimicrobials must be administered in a timely manner.^3^ Inappropriate use and over-prescribing of antimicrobials causes patient harm and may lead to increased antimicrobial resistance (AMR), which has serious consequences at an individual patient and on a population level. AMR puts at risk the ability to treat infections, resulting in prolonged illness, morbidity, and death, causing significant burden on the healthcare system.^4,5^ Without initiating effective antimicrobial stewardship (AMS) strategies, it is estimated that by 2050, AMR could cause up to ten million deaths per year worldwide.^6^

EDs perform a vital role in the initiation of appropriate antimicrobials, as patients who initially receive antimicrobials in the ED are usually discharged back to the community or are admitted to an inpatient bed, commonly without alteration to their initially prescribed therapy.^7^ However, there are significant barriers to appropriate antimicrobial prescribing in EDs, including time constraints, diagnostic uncertainty, and perceived patient preferences.^8^ Despite the challenge of the fast-paced environment of the ED, there is limited data on the antimicrobial prescribing quality in EDs at a national, or even multi-site level, which is required to identify areas for coordinated quality improvement initiatives.

The objective of this study was to evaluate the quality of antimicrobial prescribing within EDs across Australia by examining existing Hospital National Antimicrobial Prescribing Survey (Hospital NAPS) data that includes assessments of antimicrobial guideline compliance and appropriateness in relation to antimicrobials prescribed for specific indications. The aim is to identify areas of prescribing that require quality improvement to inform potential AMS strategies in the ED.

## Methods

This study is a retrospective data analysis utilizing the Hospital NAPS database between 2013 and 2022. The Hospital NAPS is a standardized auditing tool utilized by hospitals to evaluate the quality of antimicrobial prescribing. The Hospital NAPS is voluntary and is conducted by public and private hospitals across Australia. Hospitals that participated in the Hospital NAPS were classified as major city, inner regional, outer regional, remote, or very remote, based on Australian Institute of Health and Welfare (AIHW) classifications.^9^ Hospitals classified as remote or very remote were grouped together as remote for this study.

Data collection and entry was performed by trained auditors from participating hospitals using a hospital-wide point prevalence or repeat point-prevalence survey methodology. Inclusion criteria were inpatients who had been prescribed antimicrobials at 8:00 a.m. on the day of the audit, including demographics, the prescribed antimicrobial, and the indication for prescription (Appendix A in supplementary material). Data was recorded for patients who were admitted to hospital and excludes patients who were prescribed antimicrobials on discharge from the ED.

The primary outcomes were guideline compliance, as assessed against either the nationally endorsed Therapeutic Guidelines^10^ (referred to as the national guidelines) or local endorsed guidelines, and appropriateness of the antimicrobial prescription, determined according to a structured assessment matrix (Appendix B in supplementary material). More comprehensive methods regarding data collection and antimicrobial appropriateness assessment can be found in the annual Hospital NAPS public reports.^11^

The twelve most commonly prescribed antimicrobials and the twelve most common indications from 2013 to 2022 were included for analysis. As referenced in the appropriateness assessment matrix, prescriptions assessed as optimal or adequate were classified as appropriate, and prescriptions assessed as suboptimal or inadequate were classified as inappropriate (Appendix B in supplementary material). Antimicrobials were categorized according to the Priority Antibacterial List (PAL) using the Access, Review, Curb, and Contain (ARCC) classification system^12^ (Appendix C in supplementary material). The reasons for inappropriateness of antimicrobial prescriptions were also identified. The data were presented descriptively and expressed as percentages. Data analysis for guideline compliance excluded prescriptions that were classified as directed therapy, where no guidelines were available, or were not assessable.

## Results

From 2013 to 2022, there were 287,935 antimicrobial prescriptions included in the Hospital NAPS data set from 652 contributing hospitals throughout Australia. Of these, 3,098 antimicrobials (1.1%) were prescribed in EDs for 2,093 unique patients from 122 hospitals (each hospital may have contributed to the data set more than once over the period). The study population had a nearly equal distribution of sex; 50.0% male (n = 1,547), 49.5% female (n = 1,534), and 0.5% identifying as other (n = 17), with a median age of 63 years (range: 0–100 years).

Over half of prescriptions were from hospitals in major cities (54.8%, n = 1,699), a third were from inner regional EDs (33.2%, n = 1027), and only 58 prescriptions were from remote EDs (1.9%). When assessing guideline compliance in relation to remoteness classification, 2,813 prescriptions were included for analysis. Inner regional EDs had the highest rate of antimicrobial prescriptions that were compliant with local guidelines or the national guidelines^10^ (67.9%, n = 637) and major cities had the lowest rate of guideline compliance (60.2%, n = 943). Remote EDs had the highest rate of appropriateness (75.9%, n = 44), while outer regional EDs had the lowest rate of appropriateness (63.4%, n = 199).

When assessing guideline compliance, 2,813 prescriptions were included for analysis, excluding prescriptions that were directed therapy, where guideline compliance was not assessable, or there were no guidelines available. Out of these prescriptions, 213 (7.6%) were compliant with local guidelines, and 1,573 (55.9%) were compliant with the national guidelines,^10^ resulting in a rate of 63.5% (n = 1,786) guideline compliance overall.

Antimicrobial prescriptions in EDs had a lower rate of guideline compliance compared to all antimicrobial prescriptions documented in the Hospital NAPS from 2013 to 2022. Overall, 232,038 prescriptions from all departments were included for analysis of guideline compliance. Out of these prescriptions, 67.4% (n = 156,308) were deemed compliant with either local guidelines or the national guidelines.^10^

### Antimicrobial prescribing quality

Appropriateness was assessed for all 3,098 prescriptions included in the analysis. Overall, 2,181 prescriptions (70.4%) were deemed appropriate, 797 (25.7%) were deemed inappropriate, and 120 (3.9%) were not assessable. Appropriateness in the ED was lower compared to overall appropriateness from all departments (72.7%, n = 209,416)

**Figure 1.**
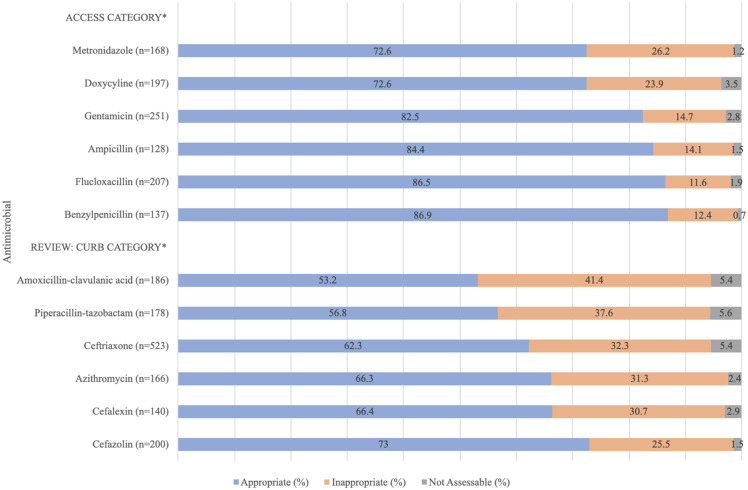
Appropriateness of antimicrobial prescriptions for the 12 most commonly prescribed antimicrobials in emergency departments from 2013 to 2022. *Antimicrobials were classified based on the Priority Antibacterial List Access, Review, Curb, and Contain classification system.^12^

The twelve most commonly prescribed antimicrobials in EDs are presented in Figure 1. These account for 80.1% (n = 2,481) of all antimicrobial prescriptions in the ED. The most commonly prescribed antimicrobials were ceftriaxone (16.9%, n = 523), gentamicin (8.1%, n = 251), and flucloxacillin (6.7%, n = 207). The antimicrobials that were prescribed with low rates of appropriateness were amoxicillin-clavulanic acid (53.2%, n = 99), piperacillin-tazobactam (56.8%, n = 101), and ceftriaxone (62.3%, n = 326) (Figure 1).

Half of the most commonly prescribed antimicrobials were classified as “Access” antimicrobials (50%),^12^ which were generally prescribed appropriately (72.6% to 86.9%) compared to those classified as “Review: Curb” antimicrobials^12^ (53.2% to 73.0%) (Figure 1).

**Figure 2.**
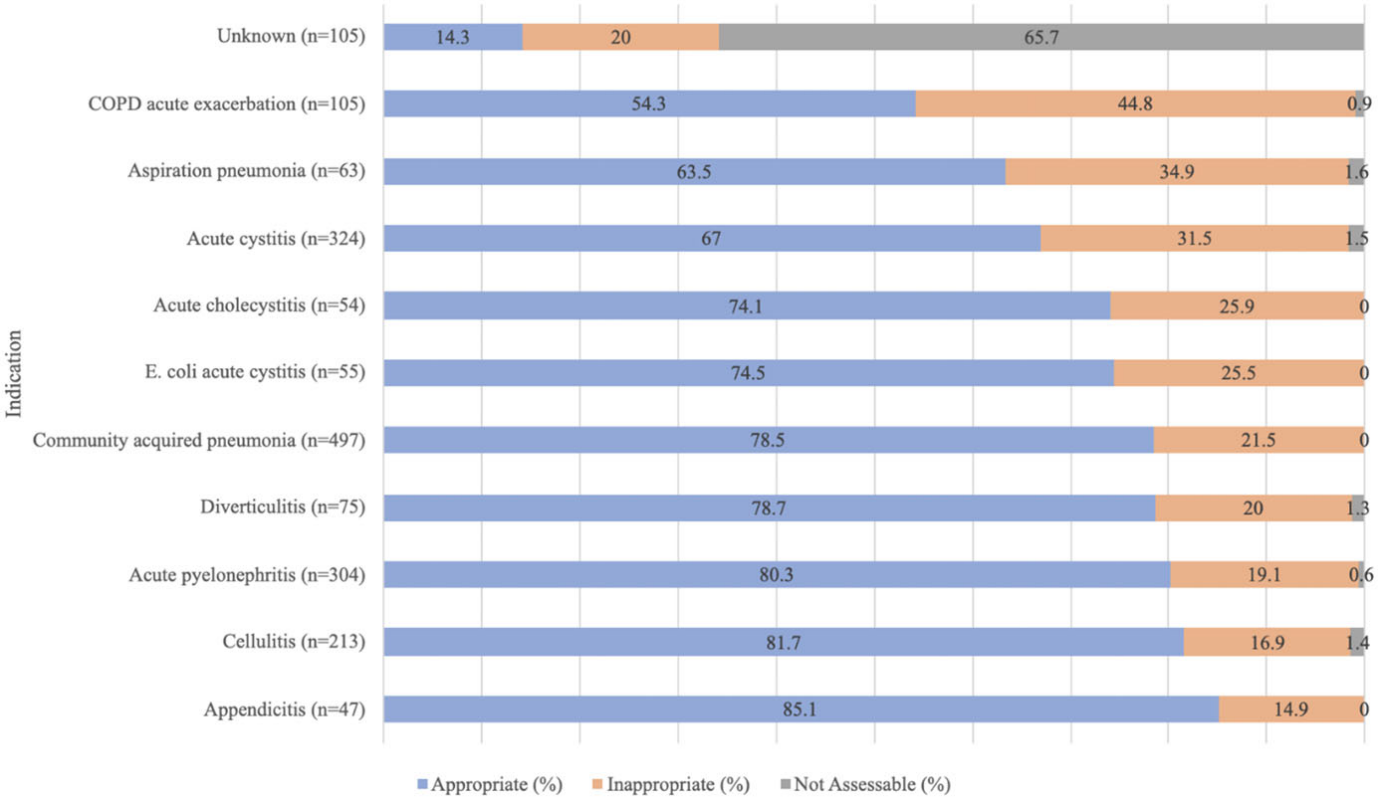
Appropriateness of antimicrobial prescriptions for the eleven most common indications in emergency departments from 2013 to 2022.

The eleven most common indications for antimicrobials in EDs are shown in Figure 2, accounting for 62.5% (n = 1,938) of all antimicrobial prescriptions in the ED. The most common indications were empiric prescribing for community-acquired pneumonia (CAP), (16.0%, n = 497), acute cystitis (10.5%, n = 324), and acute pyelonephritis (9.8%, n = 304).

The indications that had the lowest rate of appropriate antimicrobial prescriptions were acute exacerbations of chronic obstructive pulmonary disease (COPD) (54.3%, n = 57), aspiration pneumonia (63.5%, n = 40), and acute cystitis (67.0%, n = 217) (Figure 2). Ceftriaxone was the most frequent antimicrobial agent used inappropriately for COPD acute exacerbation (34.0%, n = 16), aspiration pneumonia (40.9%, n = 9), and empirically for acute cystitis (19.6%, n = 20).

**Figure 3.**
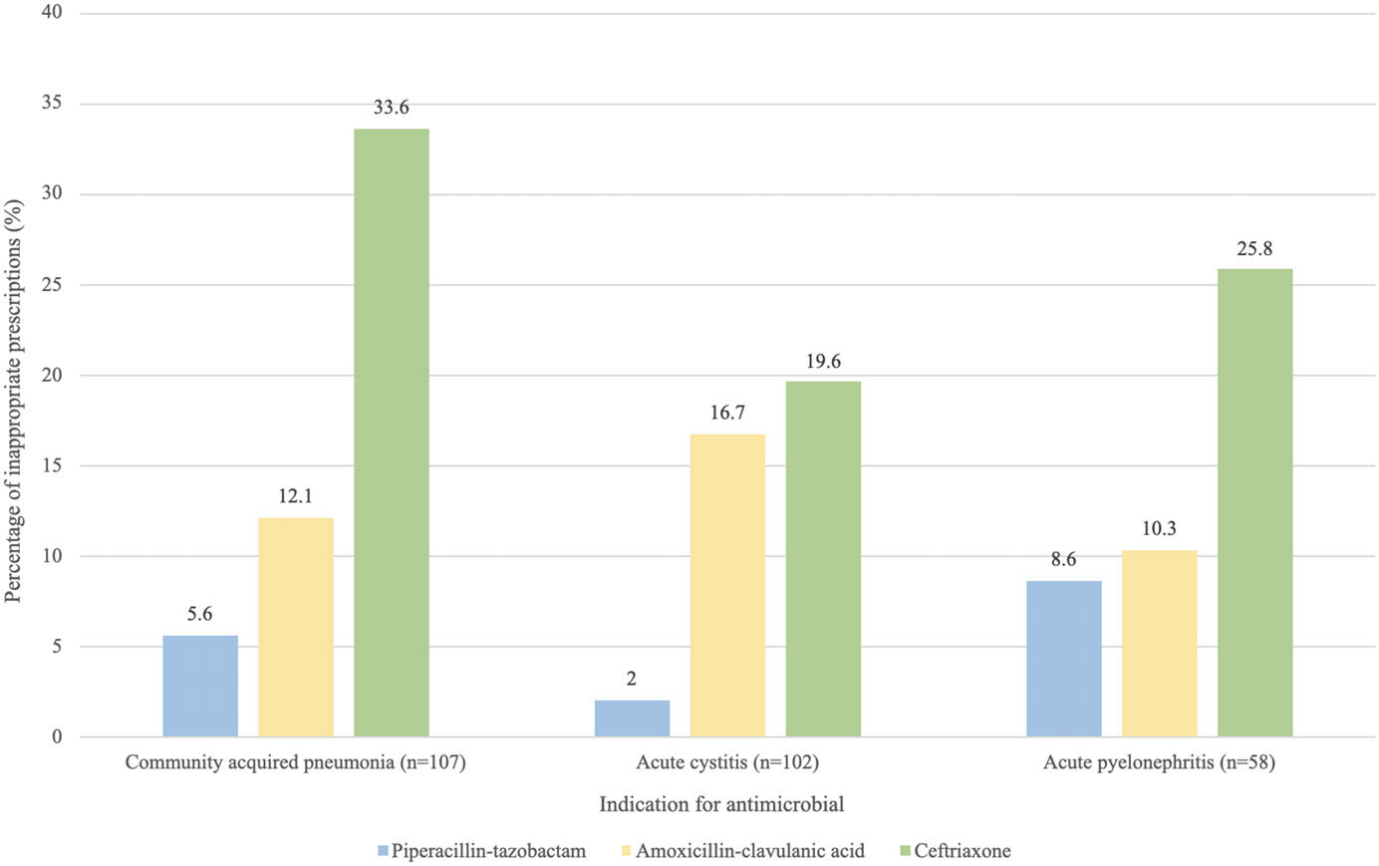
Proportion of inappropriate antimicrobials prescribed for the top three indications in emergency departments from 2013 to 2022 (n = 267).

Figure 3 presents the antimicrobials prescribed with the highest rates of inappropriateness for the most common indications in EDs. CAP was the most common indication, with 107 prescriptions deemed inappropriate, of which ceftriaxone accounted for 33.6% of all inappropriate prescriptions (Figure 3). Ceftriaxone was also the most commonly prescribed antimicrobial deemed inappropriate for acute cystitis, and acute pyelonephritis (Figure 3). There was a broad variation in antimicrobials deemed inappropriate that were used for CAP (n = 10 different antimicrobials), acute cystitis (n = 8), and acute pyelonephritis (n = 9). These accounted for 36.3% (n = 1,125) of all antimicrobial prescriptions in ED, and of which one-fifth (23.7%, n = 267) were deemed inappropriate.

**Figure 4.**
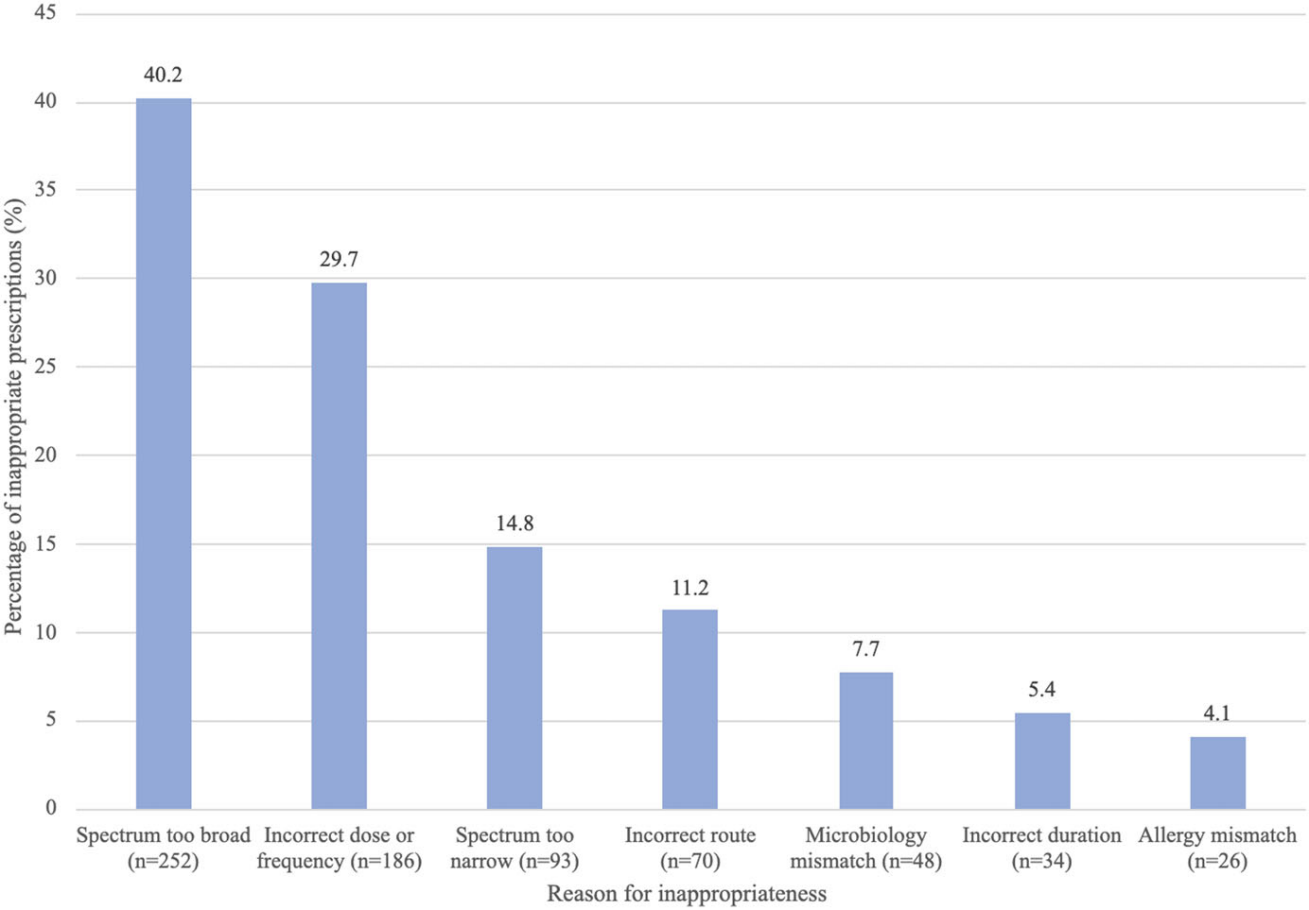
Reasons for inappropriateness for inappropriate antimicrobial prescriptions in emergency departments from 2013 to 2022 (n = 709).

Each antimicrobial prescription deemed inappropriate could have more than one documented reason for inappropriateness. Of the 797 inappropriate prescriptions, 170 (21.3%) were prescriptions where the antimicrobial was not indicated. Of the remaining 627 inappropriate prescriptions which were indicated, there were 709 documented reasons for inappropriateness. The most common reasons for inappropriateness were where the antimicrobials spectrum was deemed too broad (40.2%, n = 252), incorrect dose or frequency (29.7%, n = 186), and where the spectrum was deemed too narrow (14.8%, n = 93) (Figure 4).

The three most common documented reasons for inappropriateness for antimicrobial prescriptions deemed inappropriate are depicted in Table 1. The most common reason for inappropriateness for all three antimicrobials was spectrum too broad (59.8% for piperacillin-tazobactam, 54.4% for amoxicillin-clavulanic acid, and 41.3% for ceftriaxone) (Table 1). The most common documented reasons for inappropriateness per the common indications are similarly summarized in Appendix D in supplementary material.

**Table 1.** Reasons for inappropriateness of antimicrobial prescriptions* deemed inappropriate in emergency departments from 2013 to 2022

	Piperacillin-tazobactam (n = 62)	Amoxicillin-clavulanic acid (n = 58)	Ceftriaxone (n = 149)
Spectrum too broad	39 (62.9%)	38 (65.5%)	76 (51.0%)
Incorrect dose or frequency	11 (17.7%)	20 (34.5%)	28 (18.8%)
Spectrum too narrow	5 (8.1%)	2 (3.4%)	29 (19.5%)
Incorrect route	3 (4.8%)	3 (5.2%)	20 (13.4%)
Microbiology mismatch	7 (11.3%)	6 (10.3%)	3 (2.0%)
Incorrect duration	1 (1.6%)	5 (8.6%)	1 (0.7%)
Allergy mismatch	2 (3.2%)	3 (5.2%)	16 (10.7%)
Total Reasons for Inappropriateness	68	77	173

*n = represents the number of inappropriate prescriptions where the antimicrobial was indicated. Inappropriate prescriptions may have more than one reason for inappropriateness.

The indication with the lowest rate of appropriateness was acute exacerbation of COPD, (Figure 2). Of the 57 inappropriate prescriptions for COPD acute exacerbation, 8 (14.0%) prescriptions were deemed inappropriate as the antimicrobial was not indicated. For the 49 other inappropriate prescriptions, there were 63 documented reasons for inappropriateness with the most common reason for inappropriateness being the spectrum was deemed too broad (41.3%, n = 26).

## Discussion

This study is the first to evaluate antimicrobial prescribing quality in EDs on a national scale, where key areas that require quality improvement were determined. One area is the excessive and inappropriate use of ceftriaxone and other broad-spectrum antimicrobials, which is similar to findings from another study that evaluated antimicrobial prescribing quality in an ED in Queensland, Australia.^13^ A major finding of our study is that antimicrobials that have a high potential for resistance^12^ are commonly prescribed inappropriately, suggesting these antimicrobials a significant area to target for AMS strategies.

Ceftriaxone was the most commonly prescribed antimicrobial (16.9%, n = 523) and demonstrated one of the lowest rates of appropriateness (62.3%) in EDs. This is consistent with findings from various studies worldwide that evaluated ceftriaxone prescribing practices in EDs, which have determined appropriateness rates ranging from 47% to 62.4%.^14–17^ The Australian national guidelines^10^ indicate that ceftriaxone is not a first-line recommendation for any of the three most common indications in EDs. Specifically, ceftriaxone is not recommended for low- and moderate-severity CAP, as most cases of CAP in Australia are penicillin-susceptible.^10^ Additionally, the recommended empirical antimicrobials for low- and moderate-severity CAP and acute cystitis^10^ are part of the “Access” group antimicrobials, while ceftriaxone is listed in the “Review: Curb” group, meaning that the recommended antimicrobials have a lower potential for AMR than ceftriaxone.^12^

We postulate that ceftriaxone was commonly prescribed inappropriately for CAP and acute pyelonephritis because it was prescribed to patients with lower-severity disease. It would have been beneficial to analyze the differences in ceftriaxone prescribing based on severity of disease, however the data collected did not differentiate between severity. Despite the limitations of the data set in this study, it has previously been reported that prescribers in EDs over-estimate the severity of CAP, leading to inappropriate broad-spectrum antimicrobial therapy, such as ceftriaxone.^16,18^ Yu et al^16^ evaluated antibiotic usage for CAP over the course of twelve-months in a community hospital ED in Canada and concluded that ceftriaxone was the most commonly prescribed antimicrobial for low- and moderate-severity CAP. Ceftriaxone was also commonly prescribed inappropriately for COPD, which was the indication with the lowest rate of appropriateness (54.3% appropriate). According to the national guidelines,^10^ exacerbations of COPD only require antimicrobials when the exacerbation is severe enough to require admission to the intensive care unit, and ceftriaxone is not one of the recommended antimicrobial agents for any patient scenario. This suggests that management of COPD exacerbations is another area where AMS strategies should be implemented.

Inappropriate and excessive ceftriaxone use has significant long-term consequences for patients and potentially increases the development of AMR.^12,19,20^ Firstly, bloodstream infections caused by vancomycin-resistance enterococci (VRE) is a life-threatening nosocomial infection that has been linked to the use of ceftriaxone.^19^ Additionally, Meletiadis et al^20^ determined that patients treated with ceftriaxone had significant amplification of AMR genes in their gastrointestinal (GI) tract flora. The potential consequences of inappropriate and excessive use of ceftriaxone are severe and AMS interventions should prioritize reducing the use of ceftriaxone in EDs.

Of the most commonly prescribed antimicrobials, those with high potential for resistance (“Review: Curb” category) were generally prescribed less appropriately (53.2% to 73.0%) than those that have low potential for resistance (“Access” category) (72.6% to 86.9%).^12^ “Review: Curb” category antimicrobials are indicated as first-line treatments for many indications in the Australian national guidelines,^10^ however, the PAL identifies these antimicrobials as high-risk for AMR and suggests that the use of these agents must be closely monitored and should only be used when specifically indicated.^12^ Our data indicates that these high-risk antimicrobials are frequently used when they are not indicated, which emphasizes the importance of educating ED prescribers about which antimicrobials should be used with extra caution.

There are current clinical care standards and protocols that exist to aid in appropriate antimicrobial prescribing in Australia. The clinical care standards for AMS provide clinicians with quality statements that describe the care they should provide to patients with infections.^21^ Statements that are especially relevant for ED clinicians include the prompt treatment of patients with life-threatening conditions such as sepsis, the correct use of the national guidelines,^10^ and microbiological testing and review of therapy once investigation results are received.^21^ Importantly, the standards advise that broad-spectrum antimicrobials must be de-escalated to a more narrow-spectrum agent to reduce the potential of developing AMR.^23^ This point is of particular concern, as many patients are discharged from ED or admitted to hospital from ED without a review or change to the antimicrobial that was initially prescribed.^6^ It would have been beneficial to analyze how often the prescribed broad-spectrum antimicrobials were changed to a more narrow-spectrum agent once culture results were reviewed. However, our data shows that there was consistently over-use of antimicrobials with too broad of a spectrum in the ED. This should be an area for future research, as the unnecessary use of broad-spectrum antimicrobials contributes significantly to AMR and causes adverse drug reactions, threatening patient safety.^12,19,20^

Despite the existence of these care standards and protocols, our data shows that appropriateness of antimicrobial prescribing in EDs remains poor, especially involving the excessive and inappropriate use of antimicrobials with high potential for resistance.^12^ AMS programs have a vital role in improving the use of these antimicrobials, however the fast-paced setting of the ED makes the implementation of AMS programs more challenging.^22^ To our knowledge, there have only been two systematic reviews that have evaluated AMS strategies in EDs, which have found that there are a lack of high-quality studies on AMS interventions in EDs.^23,24^ Losier et al^23^ determined that AMS interventions may improve patient outcomes, including prospective audit and feedback, clinical guidelines, and the use of multidisciplinary teams, including pharmacists; however the optimal combination of interventions was unclear. Additionally, May et al^24^ concluded that some strategies that may improve prescribing quality include multifaceted interventions, clinical guidelines, and behavioral approaches to target prescriber behavior. With the continual over-prescribing of broad-spectrum antimicrobials with high potentials for AMR in EDs, more high-quality studies that evaluate AMS interventions are required.

There are a few limitations of this study that should be considered when interpreting this data. Firstly, sampling and selection bias are limitations of the data collection as participation in the survey is voluntary, and findings may not be representative of all Australian EDs., Almost two thirds of all Australian hospitals contribute data to the program, with 122 including data from their EDs and provides a reasonable sample size. The point prevalence methodology allows for only inpatient prescriptions to be audited. This is a limitation when analyzing overall prescription quality in the ED as many antimicrobials are prescribed upon discharge and thus excluded from the NAPS program. Further research that comprehensively assesses all antimicrobial prescriptions within the ED is warranted to develop a greater understanding of the challenges and opportunities for AMS in the ED. Another important limitation is that assessments of appropriateness and guideline compliance have some level of subjectivity. To mitigate this, auditors receive training and are guided by a structured assessment matrix.^11^

In conclusion, this study demonstrates that antimicrobial prescribing quality in EDs is suboptimal. Analysis of the Hospital NAPS data set was useful in identifying areas to target AMS interventions to improve prescribing quality. We identified that ceftriaxone and other broad-spectrum antimicrobials were excessively and inappropriately prescribed, even though clinical care standards for AMS advise the use of the most effective narrow-spectrum antimicrobial.^21^ Additionally, it was determined that antimicrobials with a high potential for AMR were prescribed less appropriately than antimicrobials with a low potential. These are areas that should be targeted for AMS interventions. We recommend that further studies should evaluate how often antimicrobials are changed after review of microbiology testing. Future research is needed to determine which AMS interventions are optimal to improve prescribing practices in EDs to decrease AMR and improve patient safety.

## Supporting information

Zosky-Shiller et al. supplementary material 1 Zosky-Shiller et al. supplementary material

Zosky-Shiller et al. supplementary material 2 Zosky-Shiller et al. supplementary material

Zosky-Shiller et al. supplementary material 3 Zosky-Shiller et al. supplementary material

Zosky-Shiller et al. supplementary material 4 Zosky-Shiller et al. supplementary material

## Data Availability

Due to the nature of the research and due to ethical requirements, supporting data is not available.

## Supplementary material

To view supplementary material for this article, please visit https://doi.org/10.1017/ash.2024.483

## References

[1] Goto T , Yoshida K , Tsugawa Y , Camargo Jr CA , Hasegawa K. Infectious disease-related emergency department visits of elderly adults in the United States, 2011-2012. J Am Geriatr Soc 2015;64:31–36. 26696501 10.1111/jgs.13836

[2] Ikuta K , Swetschinski L , Aguilar G , et al. Global mortality associated with 33 bacterial pathogens in 2019: a systematic analysis for the Global Burden of Disease Study 2019. Lancet 2022;399:629–655. 36423648 10.1016/S0140-6736(22)02185-7 PMC9763654

[3] Sherwin R , Winters ME , Vilke GM , Wardi G. Does early and appropriate antibiotic administration improve mortality in emergency department patients with severe sepsis or septic shock? J Emerg Med 2017;53:588–595. 28916120 10.1016/j.jemermed.2016.12.009

[4] Tornimbene B , Eremin S , Escher M , Griskeviciene J , Manglani S , Pessoa-Silva CL. WHO global antimicrobial resistance surveillance system early implementation 2016–17. Lancet Infect Dis 2018;18:241–242. 29396007 10.1016/S1473-3099(18)30060-4

[5] Antimicrobial Resistance. World Health Organization website. https://www.who.int/news-room/fact-sheets/detail/antimicrobial-resistance. Published 2021. Accessed July 31, 2023

[6] Interagency Coordination Group on Antimicrobial Resistance, No time to Wait: Securing the future from drug-resistant infections. World Health Organization website. https://www.who.int/publications/i/item/no-time-to-wait-securing-the-future-from-drug-resistant-infections. Published 2019. Accessed July 31, 2023

[7] O’Brien AP , Rawlins MD , Ingram PR. Appropriateness and determinants of antibiotic prescribing in an Australian emergency department. Emerg Med Australas 2015;27:83–84. 25627572 10.1111/1742-6723.12346

[8] May L , Gudger G , Armstrong P , Brooks G , Hinds P , Bhat R , et al. Multisite exploration of clinical decision making for antibiotic use by emergency medicine providers using quantitative and qualitative methods. Infect Control Hosp Epidemiol 2014;35:1114–1125. 25111919 10.1086/677637 PMC4768482

[9] Rural & Remote Australians. Australian Institute of Health and Welfare website. https://www.aihw.gov.au/reports-data/population-groups/rural-remote-australians/overview. Published 2022. Accessed August 15, 2023.

[10] Therapeutic Guidelines website. https://www.tg.org.au/. Published 2023. Accessed August 9, 2023.

[11] Antimicrobial Prescribing Practice in Australian Hospitals – Results of the 2020 Hospital National Antimicrobial Prescribing Survey. Australian Government website. https://www.amr.gov.au/resources/antimicrobial-prescribing-practice-australian-hospitals-results-2020-hospital-national-antimicrobial-prescribing-survey. Published 2023. Accessed September 14, 2023.

[12] Priority Antibacterial List for Antimicrobial Resistance Containment. Australian Commission on Safety and Quality in Healthcare website. https://www.safetyandquality.gov.au/publications-and-resources/resource-library/priority-antibacterial-list-antimicrobial-resistance-containment. Published 2020. Accessed August 9, 2023.

[13] Denny K , Gartside J , Alcorn K , Cross J , Maloney S , Keijzers G. Appropriateness of antibiotic prescribing in the Emergency Department. J Antimicrob Chemother 2018;74:515–520. 10.1093/jac/dky447 PMC6337898 30445465

[14] Durham SH , Wingler MJ , Eiland LS. Appropriate use of ceftriaxone in the Emergency Department of a veteran’s Health Care System. J Pharm Technol 2017;33:215–218.

[15] Gennai S , Ortiz S , Boussat B , François P , Pavese P. Evaluation of ceftriaxone prescriptions in the Emergency Department of a University Hospital: an urgent need for improvement and alternative therapy. Eur J Clin Microbiol Infect Dis 2018;37:2063–2068. 30069616 10.1007/s10096-018-3339-y

[16] Yu J , Wang G , Davidson A , Chow I , Chiu A. Antibiotics utilization for community acquired pneumonia in a Community Hospital Emergency Department. J Pharm Pract 2020;35:62–69. 32912068 10.1177/0897190020953032

[17] Koh HP , Ambaras Khan R , Tay SL , Gill MK , Wong JY , Zainuddin MK. Appropriateness of antimicrobial prescribing in the high-burden emergency department of a tertiary hospital in Malaysia. Int J Clin Pharm 2021;43:1337–1344. 33677792 10.1007/s11096-021-01255-w

[18] Almatar MA , Peterson GM , Thompson A , Zaidi ST. Factors influencing ceftriaxone use in community-acquired pneumonia: Emergency doctors’ perspectives. Emergency Medicine Australasia 2014;26:591–595. 25381915 10.1111/1742-6723.12326

[19] McKinnell JA , Kunz DF , Chamot E , et al. Association between vancomycin-resistant enterococci bacteremia and ceftriaxone usage. Infect Control Hosp Epidemiol 2012;33:718–724. 22669234 10.1086/666331 PMC3879097

[20] Meletiadis J , Turlej-Rogacka A , Lerner A , Adler A , Tacconelli E , Mouton JW. Amplification of antimicrobial resistance in gut flora of patients treated with ceftriaxone. Antimicrob Agents Chemother 2017;61:e00473–17. 28807914 10.1128/AAC.00473-17 PMC5655041

[21] Info for Clinicians – Antimicrobial Stewardship Clinical Care Standard. Australian Commission on Safety and Quality in Healthcare website. https://www.safetyandquality.gov.au/our-work/clinical-care-standards/antimicrobial-stewardship-clinical-care-standard/info-clinicians. Published 2020. Accessed September 5, 2023.

[22] May L , Cosgrove S , L’Archeveque M , et al. A call to action for antimicrobial stewardship in the Emergency Department: approaches and strategies. Ann Emerg Med 2013;62:69–77. 23122955 10.1016/j.annemergmed.2012.09.002 PMC3872779

[23] Losier M , Ramsey TD , Wilby KJ , Black EK. A systematic review of Antimicrobial Stewardship Interventions in the emergency department. Annals of Pharmacother 2017;51:774–790. 10.1177/1060028017709820 28539060

[24] May L , Martín Quirós A , Ten Oever J , Hoogerwerf J , Schoffelen T , Schouten J. Antimicrobial stewardship in the emergency department: characteristics and evidence for effectiveness of interventions. Clin Microbiol Infect 2020;27:204–209. 33144202 10.1016/j.cmi.2020.10.028

