# Supplementary material for: Antimicrobial prescribing quality in Australian emergency departments: an analysis of the Hospital NAPS data set
Source: Antimicrob Steward Healthc Epidemiol. 2025 Jan 17;5(1):e9. doi: 10.1017/ash.2024.483 (PMC11748020; doi:10.1017/ash.2024.483)
Supplement: Zosky-Shiller et al. supplementary material 1 — Zosky-Shiller et al. supplementary material [file S2732494X24004832sup001.pdf]

HOSPITAL NAPS National Antimicrobial Prescribing Survey

GUIDANCE | NCAS

Audit date	Patient identification number	Age / date of birth	Gender	Specialty ❒ *currently in ICU / NICU*	Ward	Weight *kg*	eGFR / CrCl *ml/min*
/ /		/ /	M / F / O				

Antimicrobials

Only record the antimicrobials as prescribed at 8:00 am on the day of the audit and any surgical prophylaxis or stat doses in the previous 24 hours

For NICU patients Birth weight *kg*	Gestational age *weeks*

Start date	Antimicrobial	Route	Dose	Freq	Prescriber code#	Indication documented	Specify documented or presumed indication	Review / stop date documented	Guideline compliance (1-6)	Surgical prophylaxis > 24 hrs	Allergy mismatch	Microbiology mismatch	Indication does not require any antimicrobials	Incorrect route	Incorrect dose / frequency	Incorrect duration	Spectrum too broad	Spectrum too narrow	If restricted: approval given	Appropriateness (1-5)
/ /																				
/ /																				
/ /																				
/ /																				
/ /																				

#Maximum of 6 characters, of which there must be at least 2 numbers

Adverse drug reactions (including allergy) to antimicrobial

❒ Nil known ❒ Present ❒ Not documented

If present, specify drugs or classes and nature of allergies

Were appropriate microbiology samples collected?

❒ Yes ❒ Partially* ❒ Not applicable ❒ No ❒ Not assessable

Record the specimen type, organism, and susceptibilities if relevant

*If more than one indication or microbiological sample is required

Guideline compliance

1. Compliant with Therapeutic Guidelines
2. Compliant with locally endorsed guidelines*
3. Non-compliant with guidelines
4. Directed therapy
5. No guidelines available
6. Not assessable

*Select **Therapeutic Guidelines** if local guidelines are the same

Clinical notes or comments

❒ Renal replacement therapy given within previous 24hrs *(e.g., dialysis)*

Surgical procedure if performed

If prophylaxis given within previous 24 hrs; *include in audit*

Appropriateness

*please refer to the **appropriateness definitions** in the resources tab or in the user guide*

1. Optimal
2. Adequate
3. Suboptimal
4. Inadequate
5. Not assessable

Doc:HospitalNAPS-DCFv10;20220218
