# Supplementary material for: Antimicrobial prescribing quality in Australian emergency departments: an analysis of the Hospital NAPS data set
Source: Antimicrob Steward Healthc Epidemiol. 2025 Jan 17;5(1):e9. doi: 10.1017/ash.2024.483 (PMC11748020; doi:10.1017/ash.2024.483)
Supplement: Zosky-Shiller et al. supplementary material 2 — Zosky-Shiller et al. supplementary material [file S2732494X24004832sup002.pdf]

Hospital NAPS appropriateness definitions

HOSPITAL NAPS National Antimicrobial Prescribing Survey

GUIDANCE NCAS

			If endorsed guidelines are <u>present</u>	If endorsed guidelines are <u>absent</u>
**Appropriate**	**1**	**Optimal[1]**	Antimicrobial prescription follows either the Therapeutic Guidelines[2] or endorsed local guidelines *optimally*, including antimicrobial choice, dosage, route and duration[3]	The antimicrobial prescription has been reviewed and endorsed by an infectious diseases clinician or a clinical microbiologist<br>**OR**<br>The prescribed antimicrobial will cover the likely causative or cultured pathogens ***and*** there is not a narrower spectrum or more appropriate antimicrobial choice, dosage, route or duration[3] available
	**2**	**Adequate**	Antimicrobial prescription does not optimally follow the Therapeutic Guidelines[2] or endorsed local guidelines, including antimicrobial choice, dosage, route or duration[3], however, is a ***reasonable*** alternative choice for the likely causative or cultured pathogens<br>**OR**<br>For surgical prophylaxis, as above ***and*** duration[3] is less than 24 hours	Antimicrobial prescription including antimicrobial choice, dosage, route and duration[3] is not the most optimal, however, is a ***reasonable*** alternative choice for the likely causative or cultured pathogens<br>**OR**<br>For surgical prophylaxis, as above ***and*** duration[3] is less than 24 hours
**Inappropriate**	**3**	**Suboptimal**	There may be a mild or non-life-threatening allergy mismatch<br>**OR**<br>Antimicrobial prescription including antimicrobial choice, dosage, route and duration[3], is an ***unreasonable*** choice for the likely causative or cultured pathogens, including:<br>• spectrum excessively broad, unnecessary overlap in spectrum of activity, dosage excessively high or duration excessively long<br>• failure to appropriately de-escalate with microbiological results	
	**4**	**Inadequate**	Antimicrobial prescription including antimicrobial choice, dosage, route or duration[3] is ***unlikely*** to treat the likely causative or cultured pathogens<br>**OR**<br>The documented or presumed indication does not require ***any*** antimicrobial treatment<br>**OR**<br>There may be a severe or possibly life-threatening allergy mismatch, or the potential risk of toxicity due to drug interaction<br>**OR**<br>For surgical prophylaxis, the duration[3] is greater than 24 hours (except where local guidelines endorse this)	
	**5**	**Not assessable**	The indication is not documented and unable to be determined from the notes<br>**OR**<br>The notes are not comprehensive enough to assess appropriateness<br>**OR**<br>The patient is too complex, due to multiple co-morbidities, allergies or microbiology results, *etc.*	

[1] Taking into account acceptable changes due to the patient's weight, allergy status, renal or hepatic function, or relevant drug interactions (if this information is available)

[2] Antibiotic Expert Group. Therapeutic Guidelines: Antibiotic. Version 16 (2019), or online version

[3] Duration should only be assessed if the guidelines state a recommended duration and the antimicrobial has already been dispensed for longer than this, or if there is a clear planned 'end date' documented

Doc:nNAPS.AD.v6.1; 20190513
