# Supplementary material for: Antimicrobial prescribing quality in Australian emergency departments: an analysis of the Hospital NAPS data set
Source: Antimicrob Steward Healthc Epidemiol. 2025 Jan 17;5(1):e9. doi: 10.1017/ash.2024.483 (PMC11748020; doi:10.1017/ash.2024.483)
Supplement: Zosky-Shiller et al. supplementary material 3 — Zosky-Shiller et al. supplementary material [file S2732494X24004832sup003.pdf]

Appendix C. Priority Antibacterial List for Antimicrobial Resistance Containment

Access	Review	
	Curb	Contain
amoxicillin	amoxicillin–clavulanic acid	amikacin
ampicillin	azithromycin	aztreonam
benzathine benzylpenicillin	cefaclor	cefepime
benzylpenicillin	cefalexin	ceftaroline
chloramphenicol	cefalothin	ceftazidime
dicloxacillin	cefazolin	ceftazidime–avibactam
doxycycline	cefotaxime	ceftolozane–tazobactam
flucloxacillin	cefoxitin	colistin
gentamicin	ceftriaxone	daptomycin
metronidazole	cefuroxime	doripenem
minocycline	clarithromycin	ertapenem
nitrofurantoin	ciprofloxacin	fosfomycin
phenoxymethylpenicillin	clindamycin	imipenem–cilastatin
procaine benzylpenicillin	erythromycin	linezolid
streptomycin	fidaxomicin	meropenem
sulfamethoxazole–trimethoprim	lincomycin	moxifloxacin
tetracycline	norfloxacin	pivmecillinam
tinidazole	piperacillin–tazobactam	polymixin B
tobramycin	rifampicin	pristinamycin
trimethoprim	rifaximin	tigecycline
	roxithromycin	
	sodium fusidate	
	spiramycin	
	teicoplanin	
	vancomycin	

Reference:

Australian Commission on Safety and Quality in Health Care. Priority Antibacterial List for Antimicrobial Resistance Containment. In: ACSQHC, editor. Sydney (Australia) 2020. Published 2020. Accessed August 9, 2023. https://www.safetyandquality.gov.au/publications-and-resources/resource-library/priority-antibacterial-list-antimicrobial-resistance-containment
